# Test accuracy of loop-mediated isothermal amplification for schistosomiasis in low endemicity areas: a systematic review and meta-analysis

**DOI:** 10.1186/s40249-025-01346-0

**Published:** 2025-07-31

**Authors:** Xinjie Zhou, Jiajia Li, Jiayin Qiu, Ting Feng, Chao Lv, Wangping Deng, Robert Bergquist, Jing Xu, Shizhu Li, Zhiqiang Qin

**Affiliations:** 1 National Institute of Parasitic Diseases, Chinese Center for Disease Control and Prevention; Chinese Center for Tropical Diseases Research; National Key Laboratory of Intelligent Tracking and Forecasting for Infectious Diseases; Key Laboratory on Parasite and Vector Biology, National Health Commission; WHO Collaborating Centre for Tropical Diseases; National Center for International Research on Tropical Diseases, Ministry of Science and Technology, Shanghai, 200025, China; 2 UNICEF/UNDP/World Bank/WHO Special Programme for Research and Training in Tropical Diseases (TDR), World Health Organization, Geneva, Sweden

**Keywords:** Loop mediated isothermal amplification, Schistosomiasis, Diagnosis, Neglected tropical diseases, Molecular diagnostics

## Abstract

**Background:**

Schistosomiasis, caused by parasitic flatworms of the genus *Schistosoma*, remains a significant public health challenge in tropical and subtropical regions, affecting over hundreds of millions of people in these areas. Accurate diagnosis is crucial for effective disease control, particularly in low-endemic areas where traditional methods like microscopy are no longer effective. We aimed to evaluate the diagnostic performance of loop-mediated isothermal amplification (LAMP) for *Schistosoma* infection.

**Methods:**

Adhering to Preferred reporting items for systematic reviews and meta-analyses guidelines, we conducted a comprehensive search on 10 May 2025 across multiple databases including PubMed, Cochrane Library, Latin American and Caribbean Literature on Health Sciences, Embase, China National Knowledge Infrastructure, and Wanfang Data, using keywords such as "schistosom*", "LAMP", and "loop-mediated isothermal amplification". Based on available literature, pooled sensitivity, specificity, positive likelihood ratio (PLR), negative likelihood ratio (NLR) and 95% confidential interval (*CI*) were calculated using STATA18.0 software. Subgroup analyses and univariable meta-regression were performed to explore the source of heterogeneity. Specifically, subgroup analyses were performed by categorizing into species (*S. japonicum*, *S. mansoni*, *S. haematobium*), sample type (stool, urine, serum, snails), and DNA extraction methods to explore factors influencing test performance.

**Results:**

The study finally included 24 individual studies derived from 14 published articles. The pooled analyses of LAMP data from all included studies resulted in a sensitivity of 0.90 (95% *CI*: 0.80–0.90), specificity of 0.82 (95% *CI*: 0.60–0.93), PLR of 4.98 (95% *CI*: 2.01–12.29), NLR of 0.13 (95% *CI*: 0.06–0.26) and diagnostic odds ratio of 39 (95% *CI*: 10–158). The area under the summary receiver operating characteristic curve reached 0.93, indicating excellent diagnostic performance. Subgroup analyses revealed optimal performance for *S. japonicum* and snail samples with lower heterogeneity (*I*^*2*^ < 50%).

**Conclusions:**

LAMP shows promise as a rapid, sensitive and specific diagnostic tool for schistosomiasis, particularly in resource-limited settings. This technique enables field application, supporting global efforts toward elimination of schistosomiasis by 2030.

**Graphical Abstract:**

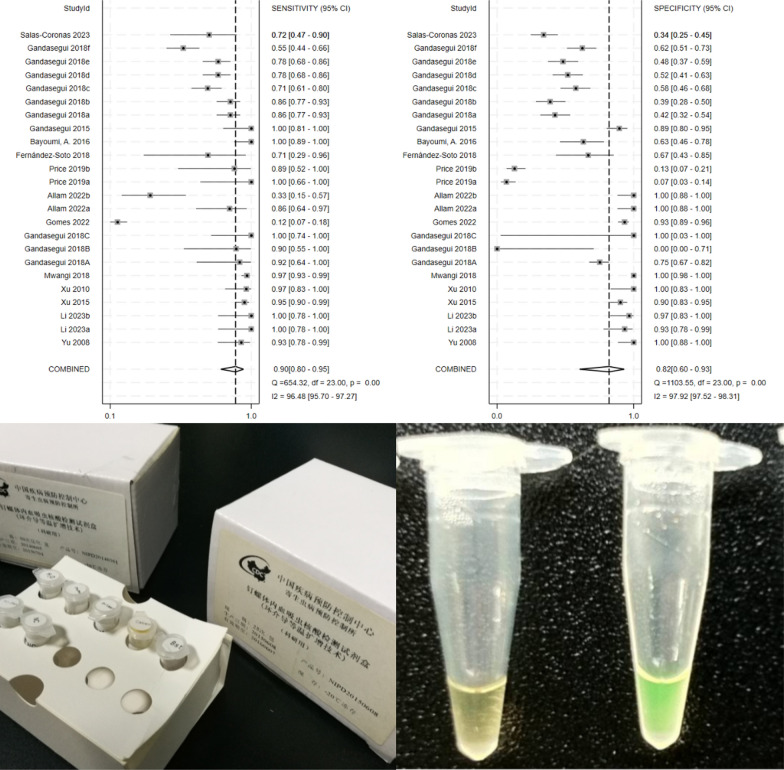

**Supplementary Information:**

The online version contains supplementary material available at 10.1186/s40249-025-01346-0.

## Background

Schistosomiasis, a disease caused by parasitic flatworms from the *Schistosoma* genus, remains a significant public health issue in tropical and subtropical regions of Africa, Asia, the Caribbean and South America [1]. It affects over 250 million people across 78 countries (primarily in Africa), causing an estimated 280,000 to 500,000 deaths annually and 3.3 million disability-adjusted life years each year [2]. Three main species of schistosomes infect humans: *S. mansoni, S. haematobium and S. japonicum* and two minor ones such as *S. intercalatum* and *S. mekongi*, while animal schistosomes and hybrid forms occasionally also infect humans [1, 3]. Infection occurs through freshwater water contact allowing exposure to schistosome cercariae—the infectious stage of the parasite released from the infected intermediate snail host [2]. The cercariae penetrate the skin of mammalian hosts, where they grow into schistosomula that eventually become adult worms [2, 4]. In intestinal schistosomiasis (caused by the majority of the species), the worms reside inside the capillary network around the middle part of the gut, and in the urogenital form of the disease (caused by *S. haematobium*) around the bladder [2, 4]. The eggs are destined to be excreted into nature with faeces or urine to infect snails, but a large part are stranded in the body where injury is caused through immunological reactions, which in intestinal schistosomiasis take place in the liver where the eggs commonly end up, or in the urogenital form in the genital organs or the bladder resulting in kidney injury due to obstructing the urine flow [2, 4]. Acute schistosomiasis, most often seen in *S. japonicum* infections, may present as self-limiting hypersensitivity reactions, known as Katayama fever [5, 6]. Chronic infection is the major manifestation where intestinal schistosomiasis is characterized by abdominal pain and diarrhoea, with long-term complications including hepatic fibrosis with potential portal hypertension eventually leading to splenomegaly and bleeding from parallel blood flow through oesophageal varicose veins [2]. Urogenital schistosomiasis presents with dysuria and haematuria, potentially leading to renal failure and squamous cell carcinoma of the bladder. Due to the potential ectopic migration of schistosome eggs or larvae, other organs such as the central nervous system and respiratory tract may also be involved [2, 3].

Even if mass drug administration with praziquantel without individual diagnosis is the most important part of current control activities [1, 2], accurate and timely diagnosis is crucial for elimination. The reason is that a general idea of schistosomiasis presence will always be needed, which is becoming increasingly difficult in areas of low endemicity where infection rates are lower, and clinical symptoms subtle or non-specific. For intestinal schistosomiasis, the reference standard for diagnosing schistosome infection is microscopic detection of eggs in stool samples using the Kato-Katz thick smear technique [2, 7, 8]. For urogenital schistosomiasis, the reference standard involves egg detection in urine after urine filtration [2, 8, 9]. These “gold standard” approaches have been available for decades, largely due to their simplicity and low cost. However, it is commonly recognized that the low sensitivity of this method presents a problem in low-intensity infection scenarios, while the reliability may be affected by daily variations in egg excretion [10]. Released schistosomal proteins in the blood (and eventually in the urine), including the circulating anodic antigen and the circulating cathodic antigen [11], can sometimes show false-positive rates [12, 13] and significant variability across different batches and versions has been reported [14, 15]. Serology detection of specific antibodies has excellent sensitivity; however, its main limitation is the inability to distinguish active from former infection [8] and there is also the potential for cross-reactivity with other helminths, leading to high false positives [16]. Multiple nucleic acid amplification techniques, including the polymerase chain reaction (PCR) and isothermal amplification have been developed and used for schistosomiasis diagnosis, demonstrating superior sensitivity to conventional microscopy, particularly in low-intensity infections [17, 18]. However, PCR-based methods involve complex procedures, costly equipment, and skilled personnel, making them impractical for resource-limited areas where schistosomiasis is endemic [17]; while loop-mediated isothermal amplification (LAMP) overcomes this obstacle and shows similar sensitivity to PCR [19].

LAMP has gained recognition as an efficient and rapid method for the early detection and accurate identification of specific nucleic acids from various organisms [19] and shown promise in diagnosing various infectious diseases, e.g., SARS-CoV-2 [20–23], toxoplasmosis [24–26], malaria [27–30] and schistosomiasis [31–33]. The source of the nucleic acid to be tested for can be blood, saliva, urine, stool, tissue and even environmental samples, such as water [34]. LAMP utilizes an enzyme derived from the large fragment of *Bacillus stearothermophilus* DNA polymerase along with six specific primers to amplify eight conserved regions of the target gene. It enables DNA amplification without reliance on thermocycling or electrophoretic separation. This operational simplicity facilitates seamless integration into clinical diagnosis applications. Through the formation of a looped structure, it enables self-cycling amplification, significantly enhancing amplification efficiency [35]. All reagents are incubated in a single tube, and the isothermal amplification process generates substantial quantities of target DNA along with reaction by-products, including magnesium pyrophosphate, which enables rapid detection through either real-time turbidity monitoring or visual fluorescence assessment using SYBR Green I/Calcein-based colorimetric analysis [36]. Due to its rapid amplification, ease of operation, and simple detection, LAMP holds promise for clinical diagnostics and infectious disease surveillance, particularly in resource-limited settings without requiring complex equipment or specialized personnel [19].

Recent studies have further optimized LAMP technology through lyophilized reagents [37], microfluidic chips [38], rapid simplified DNA extraction [39, 40], multiplex LAMP [41] and integration with CRISPR/Cas12a [42], enhancing its portability, practicality and applicability.

To identify diagnostic tools recommended by the World Health Organization (WHO) for effective detection of Schistosomiasis, we conducted a systematic review and meta-analysis on the diagnostic performance of LAMP. This effort aimed to support the goal of eliminating schistosomiasis as a public health problem by 2030 [43].

## Materials and methods

This review was conducted in accordance with the Preferred reporting items for systematic reviews and meta-analyses (PRISMA) guidelines [44] and registered in the International Prospective Register of Systematic Reviews (PROSPERO, no. CRD42025637486) to ensure that the protocol was publicly available prior to the analysis.

### Search strategy

We searched PubMed, Cochrane Library, Latin American and Caribbean Literature on Health Sciences, Embase, China National Knowledge Infrastructure and Wanfang Data as of 10 May 2025 using of the search terms: “schistosom*”, “LAMP”, “loop-mediated isothermal amplification”, etc. A detailed description of the search strategy is available as supplementary information (see S1 Appendix). No limitations were set for language, survey, or reference type. The studies we retrieved were imported into Endnote X9 (Clarivate Analytics, Philadelphia, USA) for management.

### Eligibility criteria

The studies which complied with the following criteria were included: (1) Studies analyzing human or intermediate snail host samples; (2) Minimum sample size of 10 specimens; (3) Studies using LAMP or LAMP-based assays to detect *Schistosoma* infection; (4) The reference standards comprised microscopic examination for human specimens; crushing and microscopy or cercarial shedding for intermediate host diagnosis; (5) The data of 2 × 2 tables can be extracted.

Studies containing the following were excluded: (1) inappropriate article type like editorials, reviews and conference abstracts; (2) studies using pooled snail samples; (3) duplicate publications or extended analyses of previously published data; (4) cases of schistosomal co-infections; (5) composite reference standard.

### Data collection and quality assessment

The following information was obtained from the reference papers: title, publication year, author(s), country/region, sample, reference standard, LAMP applied, species, target, DNA purification method, the number of true positives (TP), false positives (FP), false negatives (FN), true negatives (TN), and the total number of samples (N).If one article contained data obtained from different LAMP techniques, sample type or *Schistosoma* species, each set was considered a separate study.

The quality and risk of bias of the included studies were evaluated using the Quality Assessment of Diagnostic Accuracy Studies 2 tool (QUADAS-2) tool [45], a recommended tool for assessing diagnostic accuracy in systematic reviews. This tool consists of eleven criteria in four sections: patient selection, index test, reference standard, flow and timing. Each section was assessed using specific questions and rated as “High”, “Unclear”, or “Low” for risk of bias. If all signalling questions within a section were answered favourably, the corresponding risk of bias for that section was considered low. RevMan 5.4 (Review Manager; The Cochrane Collaboration, Copenhagen, Denmark) was used to analyze the QUADAS-2 result.

### Statistical analysis

We used STATA 18.0 (StataCorp LLC, College Station, USA) with the bivariate-effect models for all statistical analyses and we calculated the pooled sensitivity, specificity, positive likelihood ratio (PLR), negative likelihood ratio (NLR) and diagnostic odds ratio (DOR). The accuracy of the results was assessed by the area under the summary receiver operating characteristic (SROC) curve (AUC). The publication bias was also evaluated using Deeks'funnel plot, and a *P* value of > 0.1 indicated the absence of publication bias [46]. LAMP is a binary test with either a positive or negative result, thereby precluding the possibility of threshold bias. To assess heterogeneity, we employed the Cochran's Q test and the *I*^2^ statistic. If the *I*^*2*^ statistic exceeded 50% and the *P*-value was less than 0.1, then it would indicate substantial heterogeneity among the included studies. The univariable meta-regression and subgroup analyses were conducted to investigate potential sources of heterogeneity in the diagnostic performance of LAMP for Schistosomiasis detection. Subgroup analyses were done to examine LAMP technique performance across different regions, species, samples and more. The univariate random-effects model was employed to estimate sensitivities and specificities for subgroups containing fewer than four studies, as bivariate models fail to converge with limited sample sizes. Comparison of AUC for *S. mansoni* sample classification was conducted using a z-test, with a predetermined significance level of *P* < 0.05.

## Results

### Study selection

**Fig. 1:**
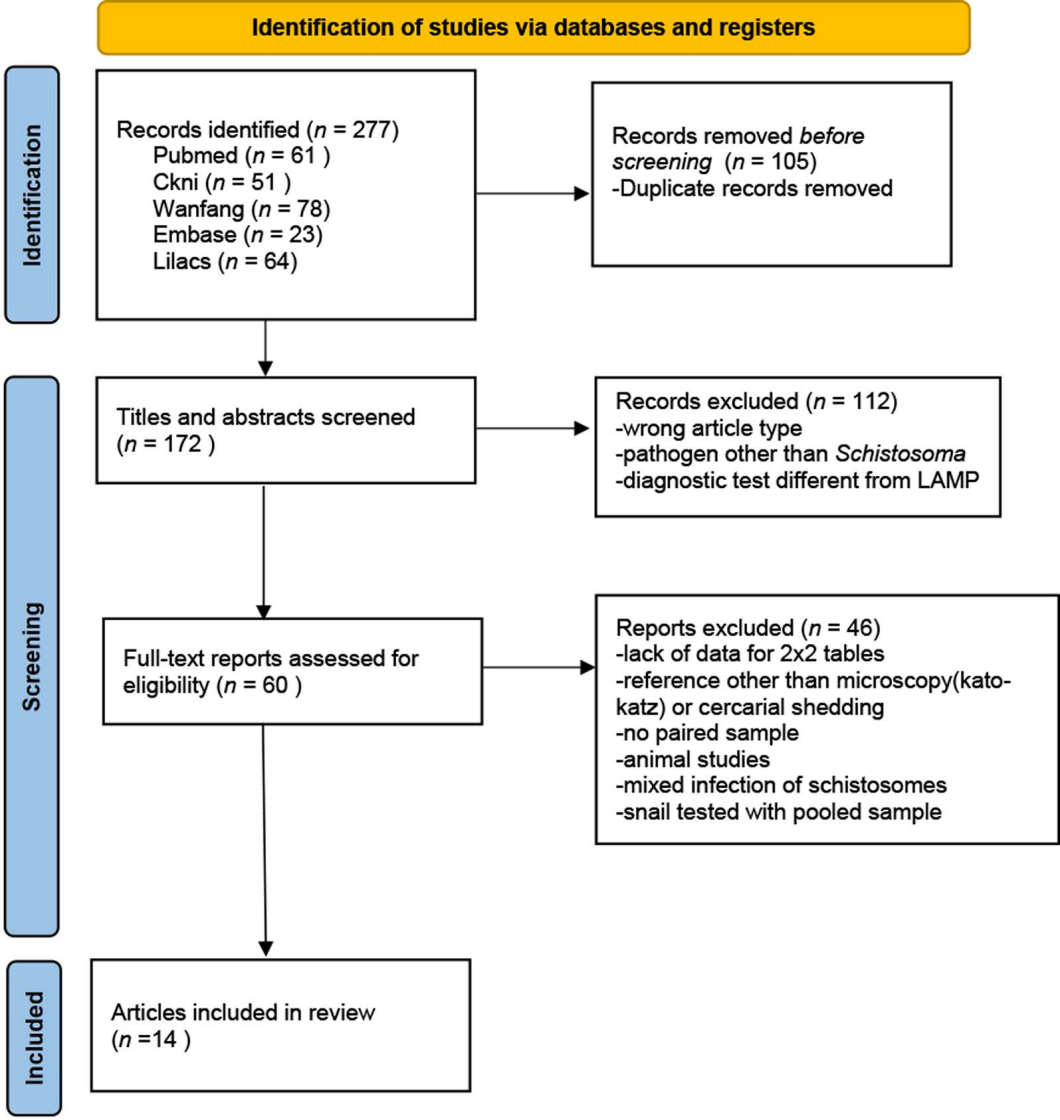
PRISMA flowchart of study selection

With the screening of titles and abstracts for all identified studies independently performed by two reviewers, a total of 277 records were initially identified through the database searches. After removing 105 duplicates, 46 records were excluded due to incompatible article/research types, while an additional 66 were eliminated based on irrelevant research subjects and/or methods. Following full-text review, 46 more records were excluded leaving 14 articles, 24 studies and 2,962 test samples. A flowchart of the research process is shown in Fig. 1. The data were extracted from these 14 articles, which encompass studies conducted across various countries categorized by species into (i) *S. japonicum*, (ii) *S. mansoni* and (iii) *S. haematobium*. Studies on *S. mekongi* and *S. intercalatum* were excluded due to insufficient amount of available research. The collected samples included (i) human serum, (ii) stool, (iii) urine and (iv) snails. The reference standards employed were classical microscopic egg detection or cercarial shedding methods. Based on the sample sources, the data were further divided into (i) human and (ii) snail categories. Additionally, to explore potential factors that could optimize LAMP detection performance and to investigate the causes of high heterogeneity, we documented the country/region, DNA purification methods, LAMP protocols and amplification targets (Table 1).

**Table 1:** Basic information and characteristics of the included studies

Author, year	Region	Target	Purification	Species	Sample	Reference	Detection	Method
Yu et al., [57]	China	SiR2	Phenol/chlor	*S. japonicum*	*O. hupensis*	Cercaria	SGI/Gel	Conv. LAMP
Li et al., [42]	China	SiR2	Kit	*S. japonicum*	*O. hupensis*	Cercaria	T	Conv. LAMP
Li et al., [42]	China	SiR2	Kit	*S. japonicum*	*O. hupensis*	Cercaria	RT-EG	LAMP-CRISPER
Xu et al., [58]	China	SiR2	Phenol/chlor	*S. japonicum*	Human serum	Kato-Katz	SGI	Conv. LAMP
Xu et al., [31]	China	SiR2	Phenol/chlor	*S. japonicum*	Human serum	Kato-Katz	SGI	Conv. LAMP
Mwangi et al., [34]	Kenya	Sm1-7	Kit	*S. mansoni*	Human stool	Kato-Katz	SGI/Ge1	Conv. LAMP
Gandasegui et al., [59]	Brazil	SmMIT	Phenol/chlor	*S. mansoni*	Human stool	Kato-Katz	SGI/Ge1	SmMIT-LAMP
Gandasegui et al., [59]	Brazil	SmMIT	Phenol/chlor	*S. mansoni*	Human stool	Kato-Katz	SGI/Ge1	SmMIT-LAMP
Gandasegui et al., [59]	Brazil	SmMIT	Phenol/chlor	*S. mansoni*	Human stool	Kato-Katz	SGI/Ge1	SmMIT-LAMP
Gomes et al., [60]	Brazil	SmITS1	Phenol/chlor	*S. mansoni*	Human stool	Kato-Katz	SGI	SmITS1-LAMP
Allam et al., [51]	Egypt	Sm1-7	Kit	*S. mansoni*	Human stool	Kato-Katz	SGI/Ge1	Conv. LAMP
Allam et al., [51]	Egypt	Sm1-7	Kit	*S. mansoni*	Human urine	Kato-Katz	SGI/Ge1	Conv. LAMP
Price et al., [54]	Zambia	Sm1-7	Kit	*S. mansoni*	Human urine	Kato-Katz	SGI/Ge1	Conv. LAMP
Price et al., [54]	Zambia	Sm1-7	Kit	*S. mansoni*	Human urine	Microscopy	SGI/Ge1	LAMP-PURE
Fernández-Soto et al., [61]	Sub-Sahara	SmMIT	Kit	*S. mansoni*	Human urine	Kato-Katz	SGI/Ge1	SmMIT-LAMP
Bayoumi et al., [62]	Egypt	IGS	Kit	*S. haematobium*	Human urine	Microscopy	SGI	Conv. LAMP
Gandasegui et al., [40]	Sub-Sahara	IGS	LAMPellet-kit	*S. haematobium*	Human urine	Microscopy	SGI/Ge1	LAMPellet
Gandasegui et al., [63]	Cent. Angola	IGS	Kit	*S. haematobium*	Human urine	Microscopy	T	Conv. LAMP
Gandasegui et al., [63]	Cent. Angola	IGS	Kit	*S. haematobium*	Human urine	Microscopy	SGI	Conv. LAMP
Gandasegui et al., [63]	Cent. Angola	IGS	LAMPellet-kit	*S. haematobium*	Human urine	Microscopy	T	SmMIT-LAMP
Gandasegui et al., [63]	Cent. Angola	IGS	LAMPellet-kit	*S. haematobium*	Human urine	Microscopy	SGI	SmMIT-LAMP
Gandasegui et al., [63]	Cent. Angola	IGS	Kit	*S. haematobium*	Human urine	Microscopy	SGI	Conv. LAMP
Gandasegui et al., [63]	Cent. Angola	IGS	LAMPellet-kit	*S. haematobium*	Human urine	Microscopy	SGI	SmMIT-LAMP
Salas-Coronas et al., [64]	Sub-Sahara	NA	Kit	*S. haematobium*	Human urine	Microscopy	SGI	Conv. LAMP

*Sm1–7 Schistosoma mansoni* 121 base pair arranged in tandem repeated sequence, *SmMIT S. mansoni* mitochondrial mini-satellite DNA region, *SmITS1* internal transcribed spacer 1 ribosomal gene of *S. mansoni*; *IGS* ribosomal intergenic spacer DNA, *LAMP-PURE* loop-mediated isothermal amplification procedure for ultra rapid extraction, Conv. *LAMP* conventional LAMP, *SGI* SYBR Green I, *Gel* Electrophoresis, *T* turbidity; *RT-EG* real-time EvaGreen fluorescence detection

### Literature quality and risk of bias

**Fig. 2:**
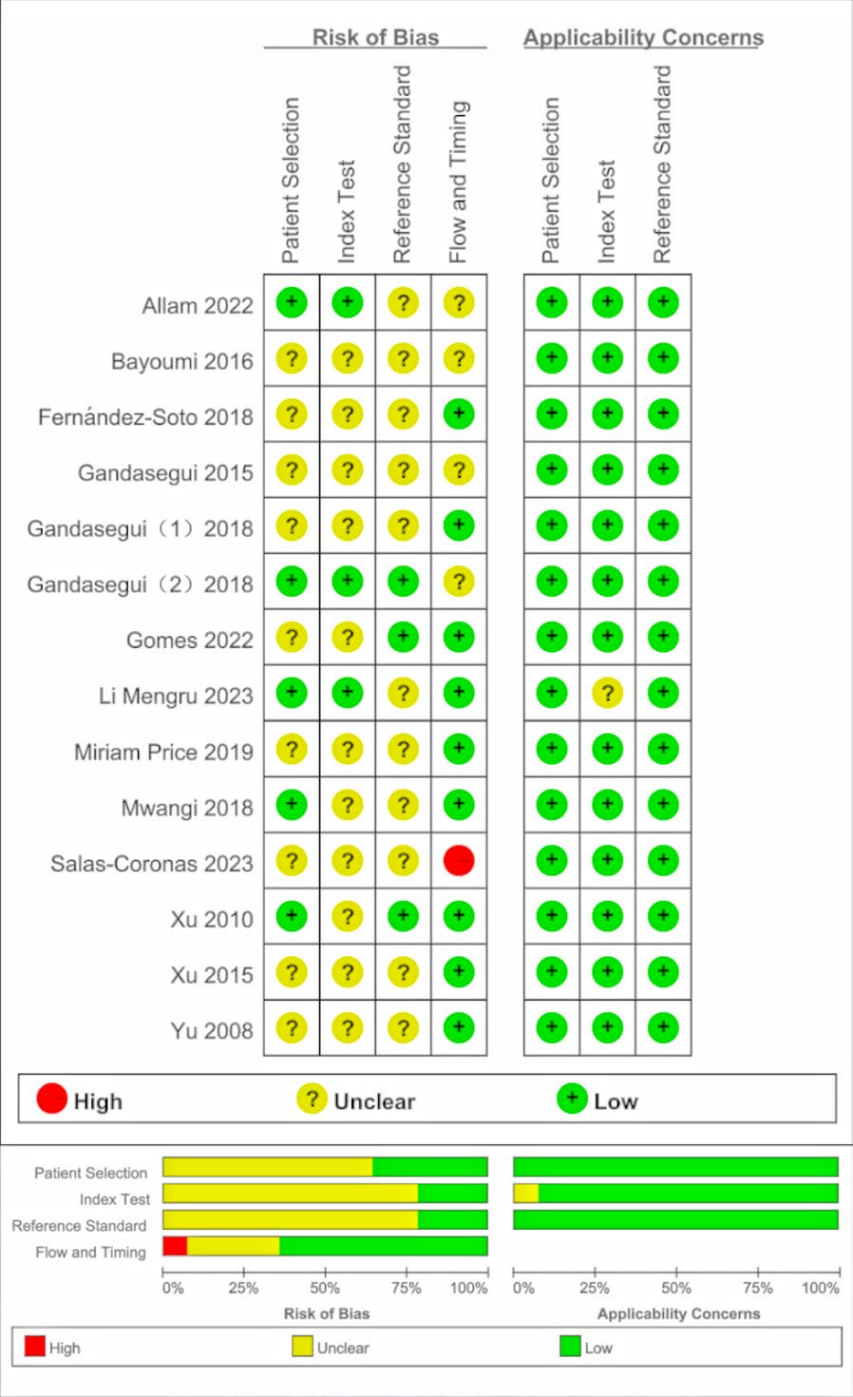
The summary of the risk of bias and applicability concerns of the included studies

There was an unclear risk of bias according to the QUADAS-2 assessment (Fig. 2). Most studies did not involve a case-control design, but the lack of clear reporting on consecutive or random sampling in Patient Selection led to an unclear risk of bias with respect to this step. Additionally, most studies did not properly apply blinding when interpreting the results of the reference standard and index test, introducing further bias in index test and reference standard. In the index test domain, the risk of bias related to the threshold effect could not be clarified, as LAMP results were binary and lack a specific positive threshold. One study had partial loss to follow-up and was marked as having a high risk of bias in Flow and Timing.

### Diagnostic performance of the LAMP assay

**Fig. 3:**
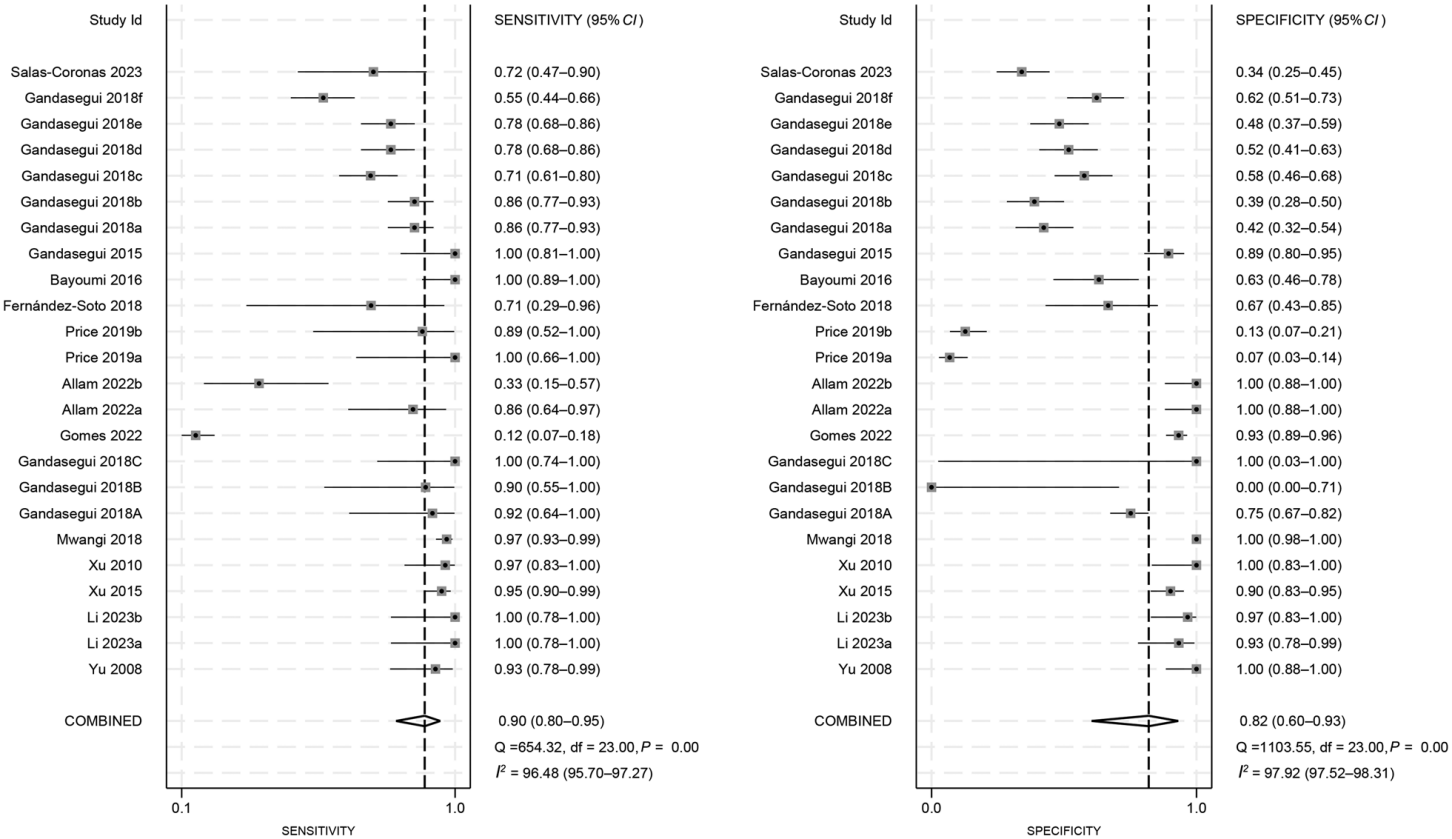
Forest plots for the pooled sensitivity and specificity of loop-mediated isothermal amplification in the diagnosis of schistosomiasis

**Fig. 4:**
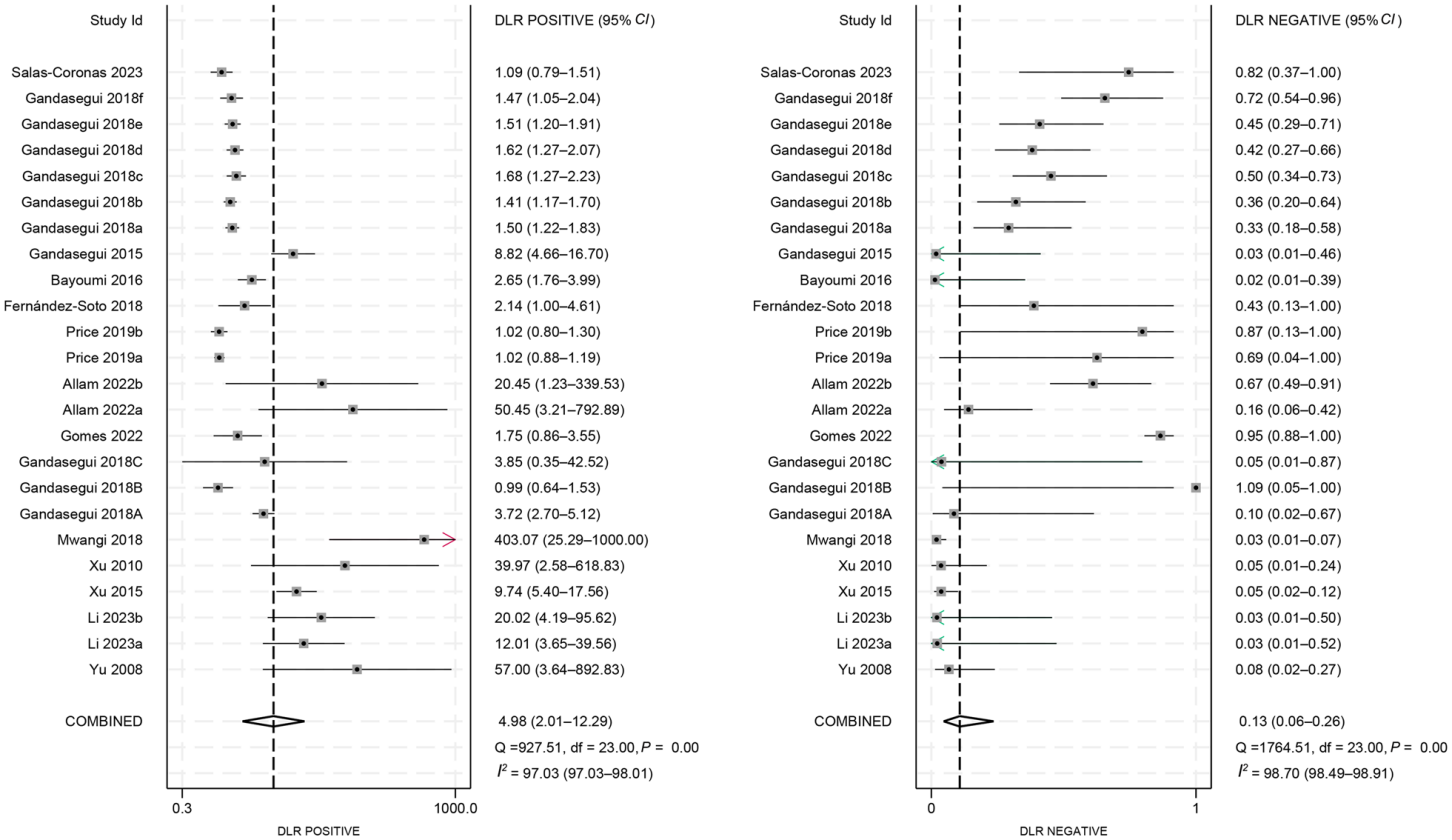
Forest plots for the pooled positive and negative likelihood ratio of loop-mediated isothermal amplification in the diagnosis of schistosomiasis

We calculated the pooled estimates, as illustrated in Figs. 3 and 4. The pooled sensitivity and specificity of LAMP were 0.90 (95% *CI*: 0.80–0.95) and 0.82 (95% *CI*: 0.60–0.93), respectively. Additionally, the pooled PLR and NLR were 4.98 (95% *CI*: 2.01–12.29) and 0.13 (95% *CI*: 0.06–0.26), respectively. The pooled DOR was 39 (95% *CI*: 10–158). Notably, significant heterogeneity was observed across the pooled estimates (*I*^*2*^ > 50%, *P* < 0.05).

**Fig. 5:**
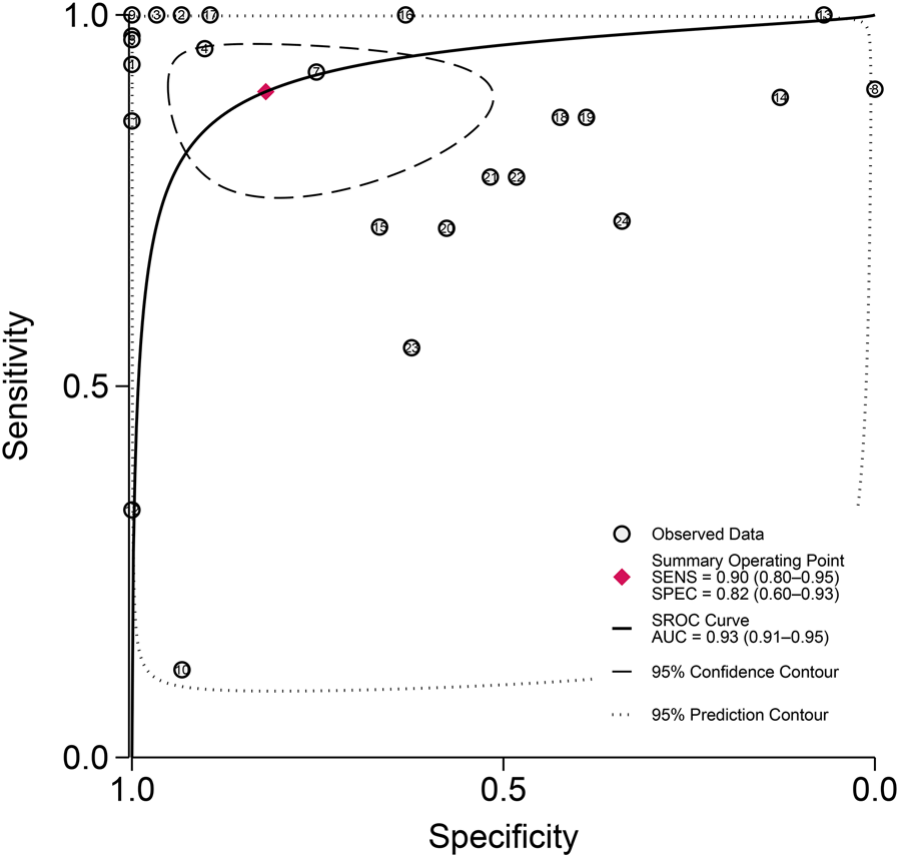
Summary receiver operating characteristic curves for loop-mediated isothermal amplification (LAMP) in the diagnosis of schistosomiasis

The accuracy evaluation results of the diagnostic techniques are presented in Fig. 5. The AUC for LAMP was 0.93 (95% *CI*: 0.91–0.95), demonstrating excellent diagnostic performance for the detection of schistosomiasis.

### Influence analysis

**Fig. 6:**
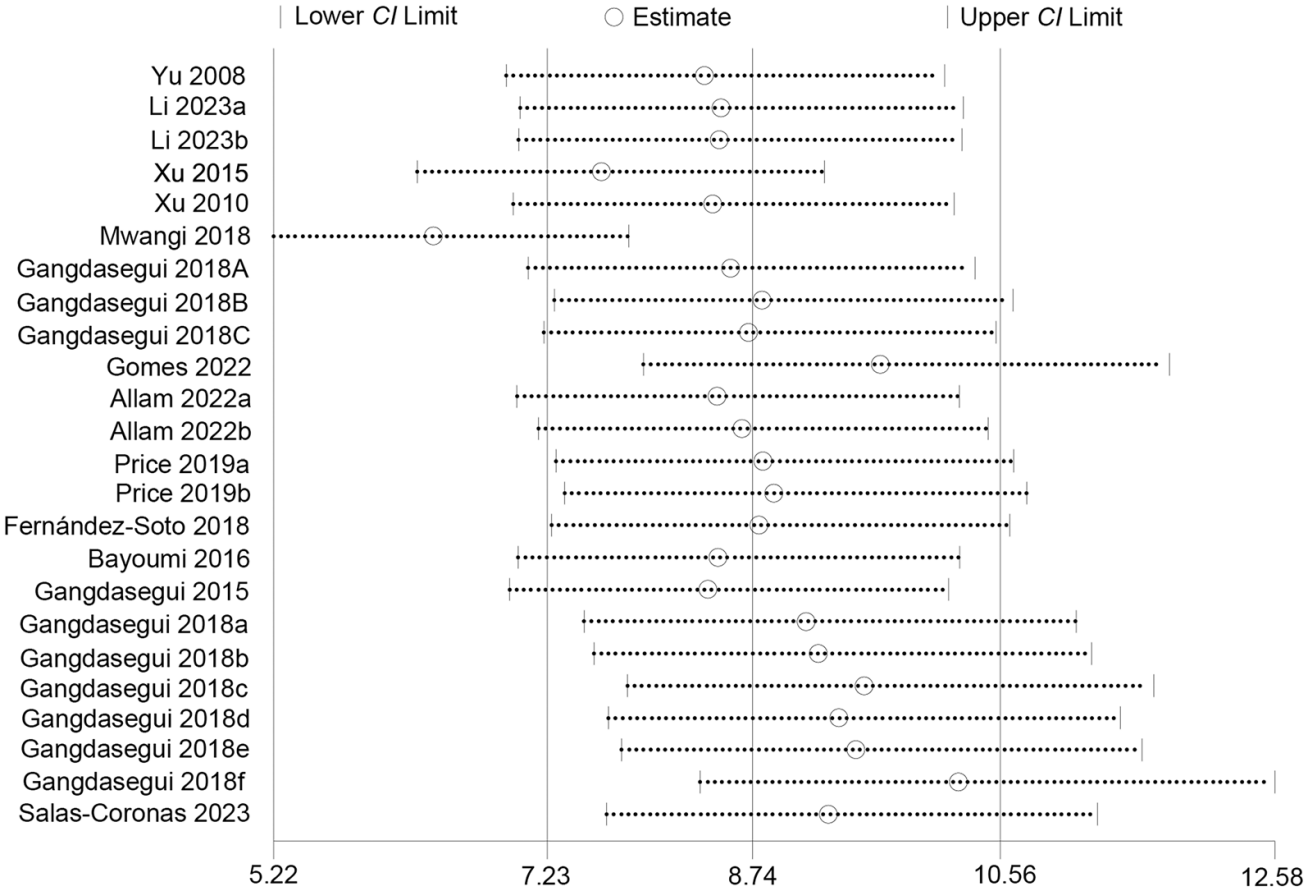
Influence analysis of the included studies

The influence analysis indicated that the included studies showed good stability, and most studies did not significantly alter the pooled DOR, with estimates ranging from 7.23 to 10.56 (Fig. 6). However, one study was identified as having a potential impact on the overall effect size of the LAMP diagnostic test. Nevertheless, even after removing this study, substantial heterogeneity persisted (*I*^2^ > 50%), suggesting that multiple other studies likely contributed to the observed heterogeneity.

### Heterogeneity analysis 

Subgroup analysis was performed to investigate potential sources of heterogeneity. As shown in Table 2, the included samples were stratified based on Purification method, Species, Sample type and Detection target. The subgroup analysis of *S. japonicum* revealed lower heterogeneity, higher pooled effect sizes and more reliable diagnostic accuracy. Similar trends were observed in subgroups involving snail samples. However, no significant differences were found within the subgroups categorized by purification methods and detection targets, and substantial heterogeneity persisted in these groups.

**Table 2:** Subgroup analysis results of loop-mediated isothermal amplification in *Schistosoma* infections

Variable	Studies included	Sensitivity (95% *CI* range) [*I*^*2*^%]	Specificity(95% *CI* range) [*I*^*2*^%]	PLR(95% *CI* range) [*I*^*2*^%]	NLR(95% *CI* range) [*I*^*2*^%]
Species					
* S. japonicum*	5	0.96 (0.92–0.98) [0.0]	0.95 (0.87–0.99) [38.7]	21.21 (6.84–65.77) [0.0]	0.04 (0.02–0.09) [0.0]
S. mansoni	10	0.87 (0.62–0.97) [96.9]	0.88 (0.30–0.99) [99.4]	7.58 (0.61–93.92) [98.2]	0.14 (0.04–0.50) [96.8]
*S. haematobium*	9	0.85(0.70–0.93) [86.6]	0.55 (0.43–0.67) [89.2]	1.90 (1.33–2.71) [76.3]	0.28 (0.12–0.65) [81.9]
Sample					
Snail	3*	0.97 (0.89–1.00) [29.6]	0.97 (0.91–0.99) [29.6]	16.76 (6.84–41.06) [0.0]	0.06 (0.02–0.18) [0.0]
Human (general)	21	0.87 (0.76–0.94) [96.1]	0.76 (0.51–0.90) [97.6]	3.61 (1.56–8.37) [95.5]	0.17 (0.08–0.35) [98.2]
Human serum	2*	0.96 (0.91–0.98) [0.0]	0.92 (0.85–0.96) [73.3]	10.85 (5.0–23.48) [7.1]	0.05 (0.02–0.11) [0.0]
Human stool	6	0.89 (0.54–0.98) [98.7]	0.98 (0.25–1.00) [99.4]	42.12 (0.32–5612.73) [99.1]	0.11 (0.02–0.63) [99.6]
Human urine	13	0.82 (0.70–0.90) [83.8]	0.53 (0.33–0.72) [94.9]	1.76 (1.17–2.63) [78.5]	0.33 (0.20–0.56) [56.0]
Purification					
Phenol/chloroform	7	0.92 (0.66–0.98) [98.7]	0.93 (0.54–0.99) [99.1]	13.09 (1.36–125.85) [98.8]	0.09 (0.02–0.45) [99.7]
Kit	13	0.90 (0.79–0.96) [90.7]	0.80 (0.43–0.96) [97.9]	4.61 (1.18–18.08) [95.9]	0.12 (0.05–0.29) [93.6]
LAMPellet	4	0.83 (0.47–0.96) [90.6]	0.68 (0.49–0.82) [90.5]	2.56 (1.19–5.53) [87.1]	0.25 (0.05–1.25) [91.9]
Detection					
SGI/Ge1	20	0.89 (0.77–0.95) [96.4]	0.83 (0.56–0.95) [8.1]	5.14(1.68–15.69) [96.9]	0.14(0.06–0.30) [98.5]
T	3*	0.80(0.74–0.86) [84.3]	0.57(0.49–0.64) [92.7]	2.11(1.23–3.61) [84.5]	0.35(0.17–0.73) 67.3]

^*^Univariate random-effects model was used to estimate sensitivities and specificities for subgroups with less than four included studies, as bivariate models do not converge when the sample size is small; *CL* confidence interval;* I*^*2*^ the proportion of the variance in observed effect due to variance in true effects rather than sampling error, *PLR* positive likelihood ratio, *NLR* negative likelihood ratio, *LAMPellet* the rapid-heat LAMPellet methodology, *SGI* SYBR green I, *Gel* electrophoresis, *T* turbidity

**Fig. 7:**
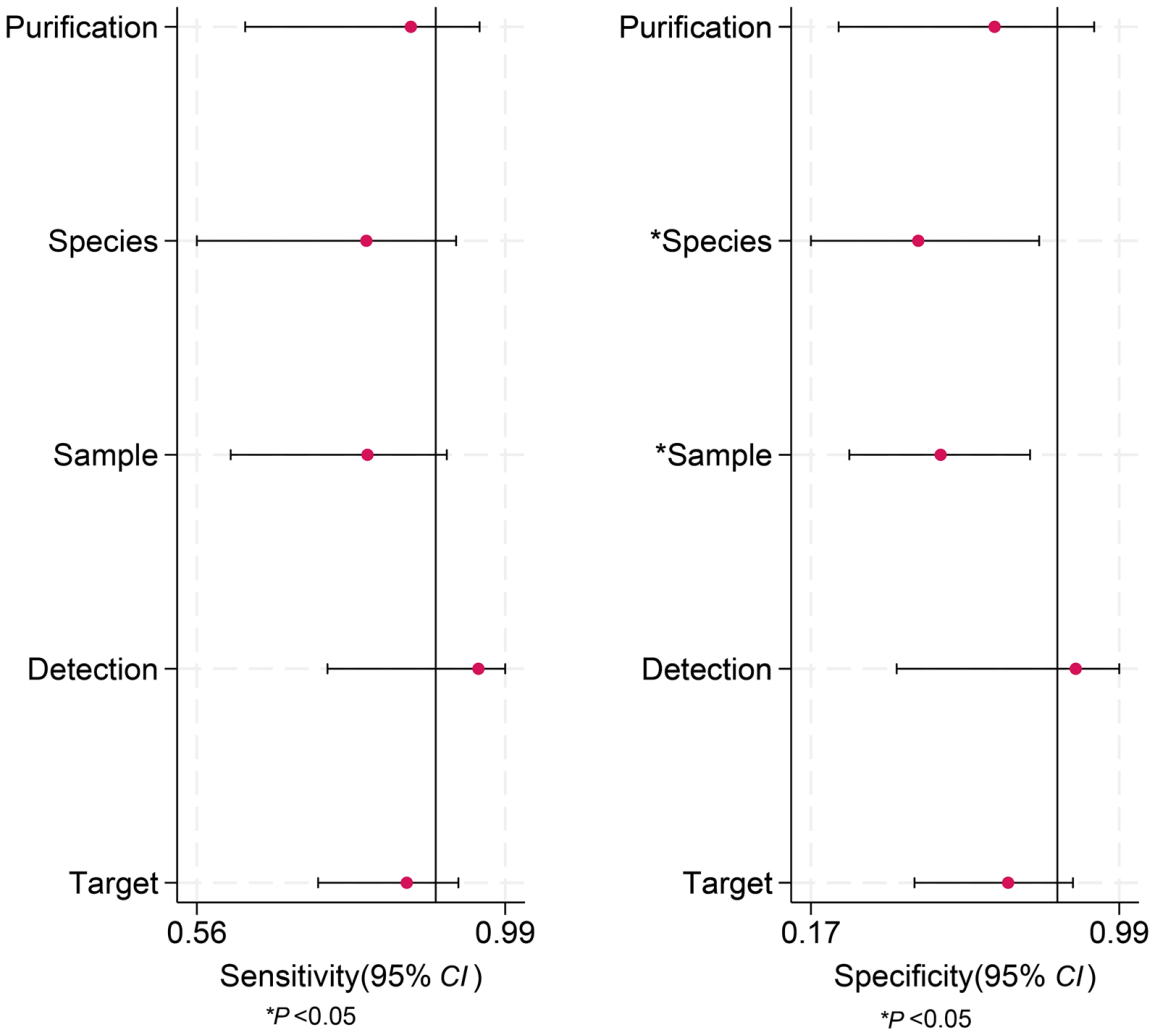
Comparison of diagnostic performance of different samples infected with *Schistosoma mansoni.* **A**: human stool sample; **B**: human urine sample

Additionally, among the included studies, *S. mansoni* was the only species with both sample types (urine and stool) and different genetic targets evaluated. Therefore, a separate analysis was conducted for this species to provide recommendations regarding sample and target selection. As shown in Fig. 7 and z-test result, the use of stool samples demonstrated significantly superior diagnostic value in LAMP for *S. mansoni* (AUC = 0.97, *Z* = 9.79*, P* < 0.001), which aligns with the pathogenic mechanism in this case. Furthermore, the *S. mansoni* 121 bp tandemly arranged repeated sequence (Sm1-7) exhibited a higher DOR compared to the *S. mansoni* mitochondrial minisatellite DNA region (SmMIT) target (Table 3). However, due to the limited number of studies and the persistent high heterogeneity within subgroups, these findings should be interpreted with caution and warrant further investigation.

**Table 3:** Subgroup analysis in *Schistosoma mansoni* infections

Variable	Studies included	Sensitivity (95% *CI* range) [*I*^*2*^%]	Specificity (95% *CI* range) [*I*^*2*^%]	PLR (95%* CI* range) [*I*^*2*^%]	NLR (95% CI range) [*I*^*2*^%]	DOR (95% *CI* range) [*I*^*2*^%]
Target						
Sm1-7	5	0.91 (0.62–0.99) [94.2]	1.0 (0.03–1.00) [99.2]	360.53 (0.03–4.5e + 06) [97.4]	0.09 (0.02–0.47) [92.7]	4097.61 (0.56–3.0e + 07) [100.0]
SmMIT	4	0.91 (0.76–0.97) [29.6]	0.73 (0.65–0.80) [67.6]	3.35 (2.53–4.43) [82.8]	0.13 (0.05–0.35) [51.1]	25.72 (8.39–78.83) [95.0]
Sample						
Stool	6	0.89 (0.54–0.98) [98.7]	0.98 (0.25–1.00) [99.4]	42.12 (0.32–5612.73) [99.1]	0.11 (0.02–0.63) [99.6]	377.03 (1.37–1.0e + 05) [100.0]
Urine	4	0.80 (0.42–0.96) [80.9]	0.53 (0.06–0.95) [97.5]	1.70 (0.48–6.03) [47.1]	0.38 (0.15–0.94) [78.9]	4.49 (0.69–29.37) [99.2]

*CL* confidence interval,* I*^*2*^ the proportion of the variance in observed effect due to variance in true effects rather than sampling error, *PLR* positive likelihood ratio, *NLR* negative likelihood ratio, *DOR* diagnostic odds ratio

**Fig. 8:**
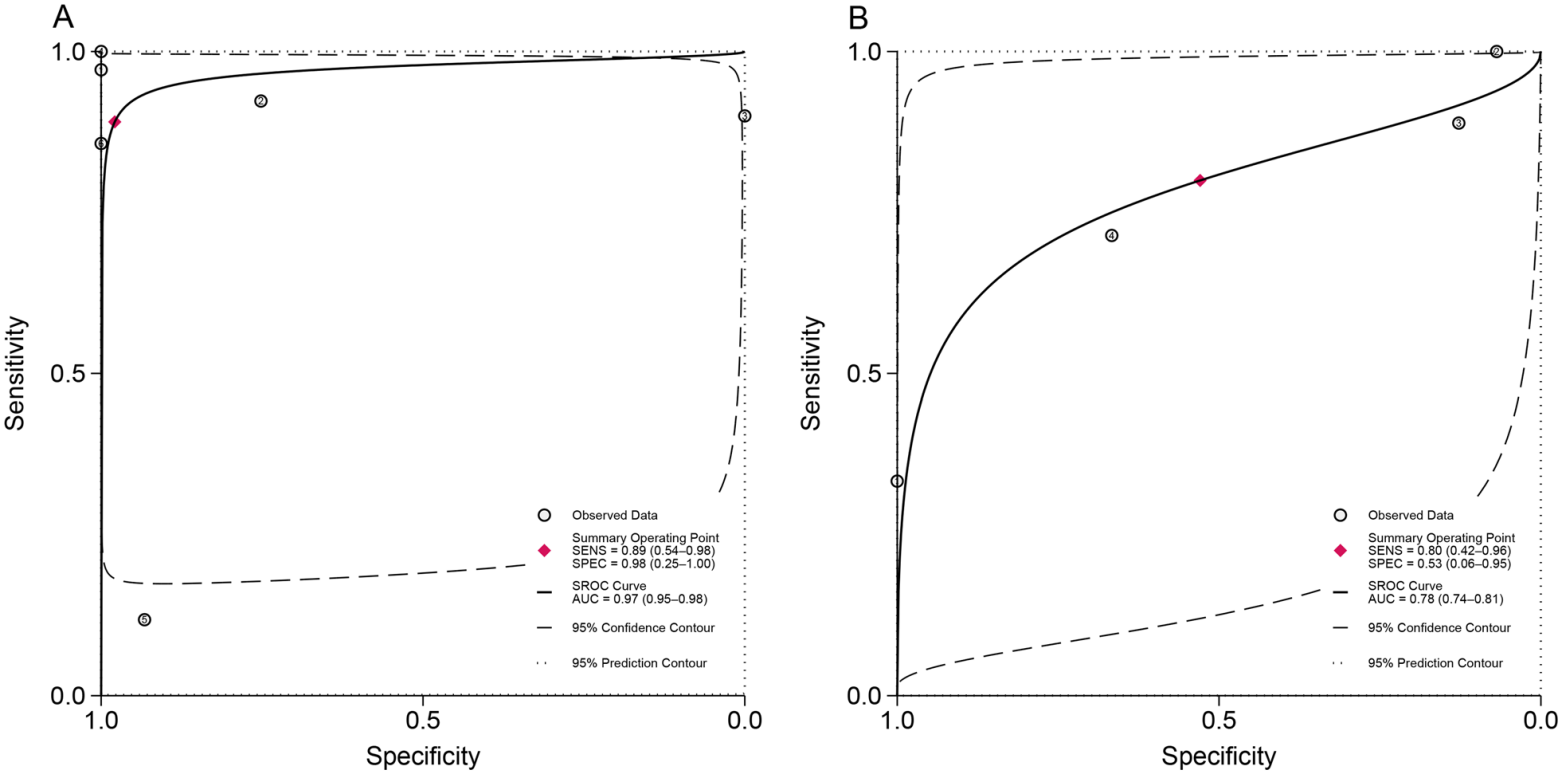
Univariable meta-regression and subgroup analyses for loop-mediated isothermal amplification (LAMP) in the diagnosis of schistosomiasis

The sensitivity and specificity of LAMP were analyzed based on various study-level covariates, including purification, species, sample type, detection method, and target gene. For sensitivity, none of the covariates demonstrated statistically significant heterogeneity, as indicated by the overlapping confidence intervals and lack of statistical significance markers. However, for specificity, species (*P* < 0.05) and sample type (*P* < 0.05) were found to be significant moderators, suggesting that differences in these factors may contribute to variability in LAMP specificity across studies. The effect estimates and confidence intervals for each covariate are visualized in Fig. 8. These findings highlight the potential impact of sample type and species variation on LAMP specificity and suggest that further standardization in diagnostic protocols may be necessary to improve test reliability.

### Publication bias

**Fig. 9:**
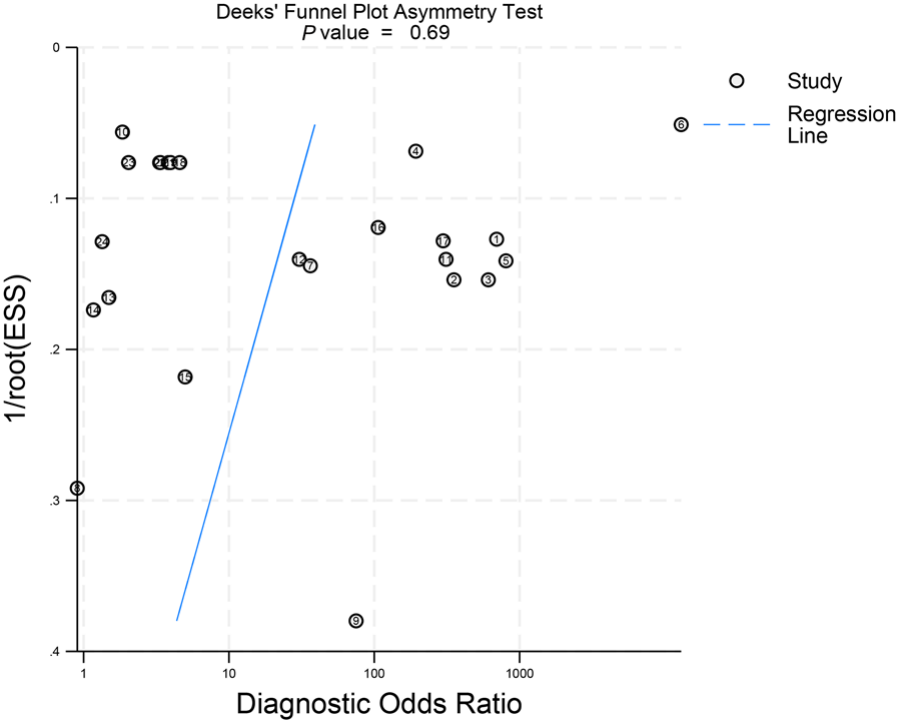
Deeks’ funnel plot of publication bias

Deeks'funnel plot asymmetry test was employed to assess potential publication bias across the included studies. The statistical analysis, as presented in Fig. 9, demonstrated no significant evidence of publication bias in the LAMP studies (*P* > 0.05).

## Discussion

Employing accurate diagnostic methods with acceptable sensitivity and specificity for proper surveillance is of utmost importance in assessing the effectiveness of all endeavours to combat schistosomiasis [8]. This study evaluated the performance of LAMP for the diagnosis of schistosomiasis, demonstrating its high diagnostic capability. LAMP technology demonstrates favorable performance in sensitivity and NLR, making it suitable as a primary screening tool to effectively exclude non-diseased individuals and reduce missed diagnoses. The PLR and DOR indicate its certain prompting role for the disease, especially in resource-limited settings where it can serve as a rapid diagnostic method.

This study for the first time conducted a comprehensive meta-analysis on the diagnostic performance of LAMP for multiple common pathogenic schistosomes. We not only quantified important indicators such as the combined sensitivity and specificity of LAMP, but also deeply explored the factors that may affect LAMP detection, providing data support for the application of LAMP in this disease and the selection of schistosome detection methods.

To explore further optimization of LAMP for the diagnosis of schistosomiasis, we analyzed the impact of various factors on LAMP performance. Subgroup regression analysis highlighted the potential influence of sample type and species variation on LAMP specificity, suggesting that further standardization of diagnostic protocols may be necessary to enhance test reliability.

The utilization of diverse sample types, particularly within the same study protocol, may represent a significant heterogeneity in diagnostic performance [47]. Previous studies have demonstrated significant disparities in detection rates between stool and serum samples when analyzed by quantitative PCR [48]. Due to the limited number of studies included, only snail and serum samples were evaluated for *S. japonicum*, with no assessment of stool samples. For *S. mansoni*, studies were available for both stool and urine samples, while for *S. haematobium*, only urine samples were examined. Among these, LAMP performance was optimal and exhibited the least heterogeneity when using intermediate host snail samples, likely due to the higher concentration of DNA extracted from snails compared to human samples such as stool and other bodily fluids. Studies have demonstrated that somatic DNA fragments from both *S. haematobium* and *S. mansoni* can be detected in urine samples [49], with extraction possible from filter paper used for urine filtration and subsequent drying, thereby eliminating the need for stool sample collection and processing [50]. In the analysis of *S. mansoni*, stool samples yielded superior results compared to urine samples. As an intestinal-dwelling schistosome, the lower concentration of cell-free DNA in urine samples may account for the reduced sensitivity of LAMP in urine-based detection [51].Additionally, potential contamination or DNA degradation during the freezing and storage of urine samples could further compromise the detection efficacy.

Despite being one of the most extensively studied neglected tropical diseases, the clinical implementation of LAMP for schistosomiasis diagnosis remains restricted, with current applications primarily confined to research scenarios rather than routine clinical practice [52]. It should be noted that LAMP presents several important disadvantages: unsuitability for cloning, highly constrained primer design, and elevated carry-over contamination risk [53]. To enhance field applicability and optimize DNA extraction from field samples, several innovative methods have been developed alongside conventional commercial kits, including LAMP-Procedure for Ultra Rapid Extraction (LAMP-PURE) [54, 55] and the Rapid-Heat LAMPellet assay [40]. Although the extraction process may occasionally yield false-negative results, the LAMP-PURE kit enables DNA extraction within minutes, making it highly suitable for rapid testing in field settings or resource-limited areas [54]. Notably, it yields significantly higher DNA concentrations compared to standard extraction methods [55]. The Rapid-Heat LAMPellet method simplifies DNA extraction by using a straightforward heating step directly on urine samples, eliminating the need for complex DNA purification. This cost-effective approach is particularly advantageous for large-scale screening programmes.

Our study has several limitations. Firstly, the limited number of studies not only potentially compromises the reliability of our findings but also precludes a comprehensive analysis of *S. mekongi* and *S. intercalatum*. Secondly, the high heterogeneity observed in our meta-analysis was not sufficiently explained by subgroup analysis. This persistent heterogeneity may be attributed to individual variations in patient characteristics, such as infection intensity, gender, age, sample collection methods, and researcher practices. Furthermore, the use of conventional microscopy as the gold standard might have led to an underestimation of the new method's diagnostic performance. Microscopy's limited sensitivity in low-intensity infections may result in false-negative cases, leading to misclassification of true positives as false positives in LAMP detection and consequently underestimating the new method's specificity. This misclassification also reduces the positive likelihood ratio and DOR. These results do not reflect inherent limitations of the new method but rather the gold standard's deficiencies. It is recommended to employ more sensitive alternative reference standards or combined reference standards to provide more accurate diagnostic benchmarks, mitigating the performance underestimation caused by the current gold standard's limitations. Additionally, the application of Latent Class Models [56] could provide unbiased estimates of sensitivity and specificity in the absence of a definitive gold standard. Therefore, further large-scale studies with diverse samples and species in field settings, accompanied by cost-effectiveness analyses are recommended to provide more comprehensive insights.

## Conclusions

In summary, LAMP demonstrates strong potential as a rapid, sensitive, and specific diagnostic tool for schistosomiasis in resource-limited settings. LAMP shows high diagnostic value, particularly for detecting *S. japonicum* infection and snail samples. It exhibits strong sensitivity and negative likelihood ratios, making it suitable for initial screening. This study provides a solid basis for the inclusion of LAMP technology in the WHO 2030 plan for the elimination of schistosomiasis.

## Supplementary Information

Additional file 1

## Data Availability

Not applicable.

## Abbreviations

LAMP: Loop-mediated isothermal amplification
*CI*: Confidential interval
PLR: Positive likelihood ratio
NLR: Negative likelihood ratio
DOR: Diagnostic odds ratio
SROC: Summary receiver operating characteristic
AUC: The area under the curve
PCR: Polymerase chain reaction
PRISMA: Preferred reporting items for systematic reviews and meta-analyses
QUADAS-2: Quality assessment of diagnostic accuracy studies 2 tool
LAMP-PURE: LAMP-Procedure for Ultra Rapid Extraction
SmMIT: *Schistosoma* *mansoni* Mitochondrial mini-satellite DNA region
Sm1-7: *S. **mansoni* 121 Bp tandemly arranged repeated sequence
