# Supplementary material for: Test accuracy of loop-mediated isothermal amplification for schistosomiasis in low endemicity areas: a systematic review and meta-analysis
Source: Infect Dis Poverty. 2025 Jul 31;14:78. doi: 10.1186/s40249-025-01346-0 (PMC12312377; doi:10.1186/s40249-025-01346-0)
Supplement: Supplementary file 1 — Additional file 1 [file 40249_2025_1346_MOESM1_ESM.docx]

**S1 Appendix.**

**Search strategy of electronic databases.**

In this systematic review and meta-analysis, we searched PubMed, Cochrane Library, Latin American and Caribbean Literature on Health Sciences (LILACS)，Embase, China National Knowledge Infrastructure (CNKI) and Wanfang Data as of 10 May 2025 using a broad search strategy.

Search in PubMed 61

Mesh:(((lamp[Title/Abstract]) OR (loop mediated isothermal amplification[Title/Abstract])) AND (sensitiv*[Title/Abstract] OR sensitivity and specificity[MeSH Terms] OR (predictive[Title/Abstract] AND value*[Title/Abstract]) OR predictive value of tests[MeSH Terms] OR accuracy*[Title/Abstract])) AND ("Schistosoma"[Mesh] OR "Schistosomiasis"[Mesh] OR S. haematobium[Title/Abstract] OR S. mansoni[Title/Abstract] OR S. intercalatum[Title/Abstract] OR S. guineensis[Title/Abstract] OR S. japonicum[Title/Abstract] OR S. mekongi[Title/Abstract] OR Schistosomas[Title/Abstract] OR Bilharzia[Title/Abstract] OR Bilharzias[Title/Abstract] OR Schistosomiases[Title/Abstract]OR Bilharziasis[Title/Abstract] OR Bilharzia[Title/Abstract] OR Katayama Fever[Title/Abstract] OR schistosom*[Title/Abstract])

Search in EMBASE 23

MeSH: ('schistosomiasis':ti OR 'schistosoma':ti OR 'schistosomiasis':kw OR 'schistosoma':kw)AND

('diagnostic*':ti OR 'diagnostic*':kw OR 'specificity':ti OR 'specificity':kw OR 'sensitivity':ti OR 'sensitivity':kw)AND ('loop mediated isothermal amplification'/exp OR (lamp OR LAMP OR 'loop mediated amplification'):ab,ti)

Search in Cochrane Library 0

Mesh:(schistosomiasis:ti,ab,kw OR schistosom*:ti,ab,kw) AND (lamp:ti,ab,kw OR "loop mediated isothermal amplification":ti,ab,kw) AND (diagnostic* OR specificity OR sensitivity)

Search in LILACS 64

MeSH:(schistosomiasis OR schistosom*) AND (diagnostic* OR sensitivity OR specificity) AND (lamp OR loop mediated isothermal amplification)

Search in Ckni 51

MESH： （主题：血吸虫 + 血吸虫病 + 血吸虫感染）AND（主题：lamp + 环介导等温扩增+环介导同温扩增）AND（摘要：诊断 + 价值 +准确 +敏感 +特异+ 精确 + 评价 (精确)）；检索范围：总库

Search in Wangfang 78

[MESH:(血吸虫 OR 血吸虫病 OR 血吸虫感染) and 主题:(lamp OR 环介导等温扩增 OR 环介导同温扩增) and 主题:(诊断 OR 价值 OR 准确 OR 敏感 OR 特异 OR 精确 OR 评价)](https://s.wanfangdata.com.cn/advanced-search/paper?q=%E4%B8%BB%E9%A2%98%3A(%E8%A1%80%E5%90%B8%E8%99%AB%20OR%20%E8%A1%80%E5%90%B8%E8%99%AB%E7%97%85%20OR%20%E8%A1%80%E5%90%B8%E8%99%AB%E6%84%9F%E6%9F%93)%20and%20%E4%B8%BB%E9%A2%98%3A(lamp%20OR%20%E7%8E%AF%E4%BB%8B%E5%AF%BC%E7%AD%89%E6%B8%A9%E6%89%A9%E5%A2%9E%20OR%20%E7%8E%AF%E4%BB%8B%E5%AF%BC%E5%90%8C%E6%B8%A9%E6%89%A9%E5%A2%9E)%20and%20%E4%B8%BB%E9%A2%98%3A(%E8%AF%8A%E6%96%AD%20OR%20%E4%BB%B7%E5%80%BC%20OR%20%E5%87%86%E7%A1%AE%20OR%20%E6%95%8F%E6%84%9F%20OR%20%E7%89%B9%E5%BC%82%20OR%20%E7%B2%BE%E7%A1%AE%20OR%20%E8%AF%84%E4%BB%B7)&type=%5b%22periodical%22,%22thesis%22,%22conference%22,%22patent%22,%22standard%22,%22cstad%22,%22law%22,%22nstr%22,%22localchronicleitem%22%5d&chineseEnglishExpand=true&topicExpand=true)
